# Antibacterial, Redox, Cytotoxic, and Ecotoxic Properties of New Sol–Gel Silica-Copper-Based Materials

**DOI:** 10.3390/ph19010035

**Published:** 2025-12-23

**Authors:** Iliana Ivanova, Lilia Yordanova, Lora Simeonova, Miroslav Metodiev, Elena Nenova, Deyan Monov, Yoanna Kostova, Albena Bachvarova-Nedelcheva, Iva Kirova, Elitsa Pavlova

**Affiliations:** 1 Faculty of Biology, Sofia University “St. Kliment Ohridski”, 8 Dragan Tsankov Blvd., 1164 Sofia, Bulgaria; iaivanova@biofac.uni-sofia.bg (I.I.); lilijapj@uni-sofia.bg (L.Y.); nenova@uni-sofia.bg (E.N.); deyanmoni@gmail.com (D.M.); 2 Department of Virology, Stephan Angeloff Institute of Microbiology, Bulgarian Academy of Sciences, 26 G. Bonchev Str., 1113 Sofia, Bulgaria; losimeonova@gmail.com (L.S.); metodiev.1996@abv.bg (M.M.); 3 Institute of Metal Science, Equipment and Technologies with Hydro- and Aerodynamics Centre “Acad. A. Balevski”, Bulgarian Academy of Sciences, Shipchenski Prohod Str., 67, 1574 Sofia, Bulgaria; y_kostova@ims.bas.bg; 4 Institute of General and Inorganic Chemistry, Bulgarian Academy of Sciences, Acad. G. Bonchev Str., Bl. 11, 1113 Sofia, Bulgaria; albenadb@svr.igic.bas.bg; 5 Faculty of Physics, Sofia University “St. Kliment Ohridski”, 5 James Boucher Blvd., 1164 Sofia, Bulgaria; iva_kirova88@abv.bg

**Keywords:** sol–gel technique, silica-copper-based materials, antibacterial activity, ROS, cytotoxicity, eco-safety

## Abstract

**Objective:** The objective of our study was to synthesize and characterize silica–copper nanomaterials and to evaluate their biological properties (antibacterial, redox, cytotoxic, and ecotoxic) for potential applications. **Methods and Results:** Si/Cu-based materials were prepared by a sol–gel method. They were characterized by XRD, UV-Vis, and SEM-EDS. The antibacterial activity of the materials was evaluated against Gram-positive bacteria (*Staphylococcus aureus*, *Bacillus cereus*), Gram-negative bacteria (*Escherichia coli*, *Pseudomonas aeruginosa*, *Salmonella typhimurium*), and yeasts (*Candida albicans*, *Saccharomyces cerevisiae*). The nanomaterial that was calcined at 500 °C exhibited greater antibacterial efficacy compared to the gel form. *S. typhimurium* demonstrated the highest susceptibility, whereas *S. aureus* and *P. aeruginosa* were the most resistant of the tested bacteria. Both yeasts exhibited comparable sensitivity (MBC = 1.0 mg/mL). The redox activity of both nanomaterials was tested at pH 7.4 (physiological) and 8.5 (optimal) by the activated chemiluminescent method. The nanocomposites significantly inhibited the free-radical and ROS generation. This presents them as redox regulators in living systems. The cytotoxic effects in normal BEAS-2B and tumor A549 human cell lines were assessed microscopically and by the cell viability neutral red uptake assay, CC_50_ being evaluated. The observed effects suggest moderate, similar cytotoxicity in both cell lines. The ecotoxicity study using *Daphnia magna* showed an LC_50_ of ~7–8 mg/L about Si/Cu/500. The LC_50_ for Si/Cu (gel) was lower than 0.25 mg/L, indicating an increase in toxicity with increased exposure time. **Conclusions:** Possible applications of the newly synthesized nanomaterials include antimicrobial coatings, drug delivery systems, antioxidant additives in various formulations, and water purification.

## 1. Introduction

Copper nanomaterials are widely produced due to their abundance, low cost, and versatility compared to gold and silver [1]. Among the various synthesis techniques, chemical “bottom-up” approaches, such as the sol–gel method, are commonly used, enabling the assembly of atoms or molecules into nanoscale structures [2]. The sol–gel method in particular is valued as “green” and efficient for the production of copper nanoparticles with notable mechanical, electrical, optical, and catalytic properties [3,4].

Recent advances in nanotechnology have led to extensive research on nanomaterials as potential alternatives to conventional antibiotics [5,6]. Nanoparticles are increasingly applied in antibacterial coatings for medical implants and wound dressings, in drug delivery systems, microbial detection platforms, and vaccine formulations [7]. Although their antibacterial mechanisms are not yet fully understood, they are generally attributed to oxidative stress induction, metal ion release, and non-oxidative pathways. The simultaneous action of these multiple mechanisms makes the development of bacterial resistance highly unlikely [8].

Silica (Si) nanoparticles are attracting increasing interest due to their properties and biocompatibility. Silicon is one of the fundamental materials in the human body. The silicon nanoparticles are completely degradable in the living organism [9]. They are very promising for various biomedical applications. The oxidized Si-nanoparticles, made of porous silicon, with various surface modifications, can easily penetrate living cells without causing acute cytotoxic effects. Actually, silica nanomaterials are low-toxic among the various biomedical nanomaterials [10]. The interaction of Si nanoparticles with biological systems in vitro and in vivo has been studied, and their negligible toxicity has been proven in scientific reports. Recently, it has been found that silicon nanoparticles also possess antimicrobial properties. This effect is due to their large surface area, inhibiting bacterial adhesion in order to form biofilms [11]. In recent years the combination of Si nanoparticles with other biocidal metals (e.g., Ag, Cu) has been investigated. It has been found that such materials possess strong antibacterial activity against pathogens, such as *E. coli* and *S. aureus*. Several studies have shown that silicon nanomaterials can interact with living cells and bacteria, disrupting cellular functions such as cell differentiation, adhesion, and proliferation. All these findings indicate that Si nanocomposites loaded with other antimicrobial metals have great potential to be developed as promising antimicrobial agents [12,13].

Traditionally, all newly synthesized substances are tested and characterized in detail, as special attention must be given to the materials at the nanoscale, as their properties frequently diverge from the expectations and those usually described for the compounds of the same element. The extremely small dimensions of the nanomaterials enable their enhanced physical properties. Furthermore, their high surface energy often enhances chemical reactivity [14]. Their mechanical effects on the living organism also need special attention.

When the nanomaterials are introduced into the organism, they engage with its components and metabolites. It is essential to evaluate their safety, or conversely their potential as agents with antimicrobial and/or cytotoxic properties, to apply them practically across different tasks [15,16,17].

*Daphnia magna* (Cladocera, Crustacea) is one of the most widely used and standardized test organisms in global ecotoxicology [18]. Daphnia are extremely sensitive to a wide range of pollutants. This makes them an excellent “early indicator” of potentially harmful effects on aquatic ecosystems. The life cycle, behavior, growth and metabolism of *D. magna* have been studied in detail, which provides a solid basis for interpreting toxicological data. Changes in behavior such as motility, respiratory rate (antennae movement) or avoidance of a pollutant can be easily observed, which are rapid and sensitive indicators of stress. The study aimed to expose *Daphnia magna* to Si/Cu nanoparticles to perform acute toxicity studies. Nanoparticles based on silicon and copper are widely used in various applications, and their impact on organisms is important. There is not much research on their safety for aquatic species. Such tests are fundamental for assessing the ecological risk of chemicals and for determining safe levels for aquatic ecosystems [18,19,20].

In this study, we synthesized silica–copper nanoparticles via the sol–gel method to ensure uniform dispersion and controlled particle formation. Their antibacterial activity was tested against two Gram-positive bacteria, three Gram-negative bacteria, and two yeast species. The minimum inhibitory concentration (MIC) and the minimum bactericidal concentration (MBC) were calculated. Their redox activity towards free radicals and reactive oxygen species (ROS) was examined in three model chemical systems. It provided an insight into the action mechanism underlying their biological effects. Their cytotoxicity was evaluated in tumor A549 and normal BEAS-2B epithelial cell lines. It was calculated as CC_50_. Their eco-toxicological safety was further evaluated using *Daphnia magna*—a standard freshwater indicator organism. All these assays and the obtained results illustrate their potential applications and limitations. The novelty of this work lies in the comprehensive biological evaluation of sol–gel-derived Si-Cu nanocomposites, extending beyond conventional antimicrobial assays. It integrates tests of cytotoxicity in cell lines, redox activity, and ecological safety, thereby providing an original multi-method approach. Calcination effects are well-known in materials, but direct bio-efficacy contrasts within reports on similar materials are sparse or missing. Up to now, highly sensitive chemiluminescent evaluations testing the reactivity of nanomaterials with ROS have been made only by our team. Overall, the synthesis strategy offers precise control over nanomaterials’ properties, enhancing their suitability for biomedical and environmental applications. This integrated multidisciplinary study delivers valuable perspectives on the safe and effective use of hybrid Si–Cu nanocomposites.

## 2. Results and Discussion

### 2.1. XRD and SEM Morphology Results

**Figure 1:**
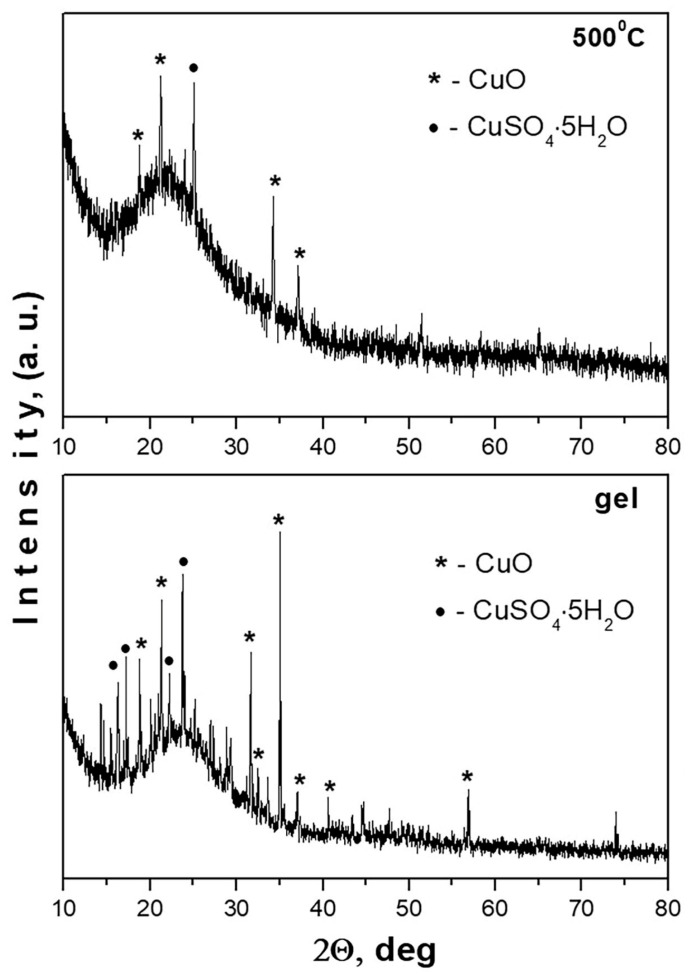
XRD patterns of the prepared hybrid Si/Cu (gel) and Si/Cu/500, heat-treated at 500 °C powder.

Figure 1 shows the XRD patterns of the gel and heat-treated samples. This analysis was used to investigate the changes in the phase formation upon heating. As is seen from the figure, the XRD pattern of the as-prepared gel exhibited an amorphous silica with sharp peaks of both crystalline phases—CuO (JCPDS 45-0937) and CuSO_4_·5H_2_O (JCPDS 72-0084). After the heat treatment at 500 °C for 2 h exposure time, the XRD showed an increase in the amorphous halo and a decrease in the number of characteristic peaks for both crystalline phases. This could be related to the successful thermal decomposition and phase transformation of CuSO_4_·5H_2_O.

**Figure 2:**
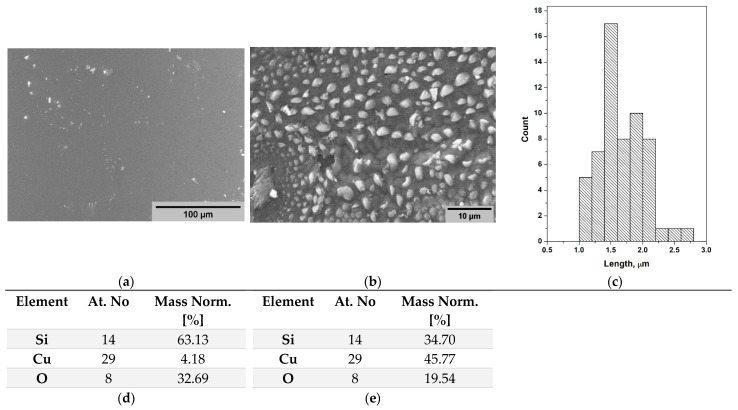
SEM micrographs of the samples: Si/Cu (gel) (**a**), Si/Cu/500, heat-treated at 500 °C (3 h) powder (**b**), histogram of the particle size distribution (**c**), elemental composition in wt% obtained by EDS analysis of the gel (**d**) and heat-treated sample (**e**).

Figure 2a,b displays the SEM images of the investigated samples. The image of Si/Cu (gel) shows a nearly smooth surface. The heat-treated at 500 °C sample presents a surface covered with irregularly shaped particles, distributed randomly. The histogram of the particle size distribution is shown in Figure 2c, and it shows that the average particle size is about 1.5–2.0 μm. The EDS analysis revealed the presence of silica and copper components in both samples, as shown in Figure 2d,e. As is seen from the figure, there is an apparent increase in the Cu concentration (from 4.18 to 45.77 wt%) and a corresponding decrease in Si and O from the substrate after heating the sample in air. This indicates that Cu diffused to the surface and oxidized, forming a Cu-rich oxide layer that covered part of the SiO_2_ substrate. The result is in good agreement with those obtained by other authors [21,22].

### 2.2. UV-Vis Spectral Analysis of the Samples

**Figure 3:**
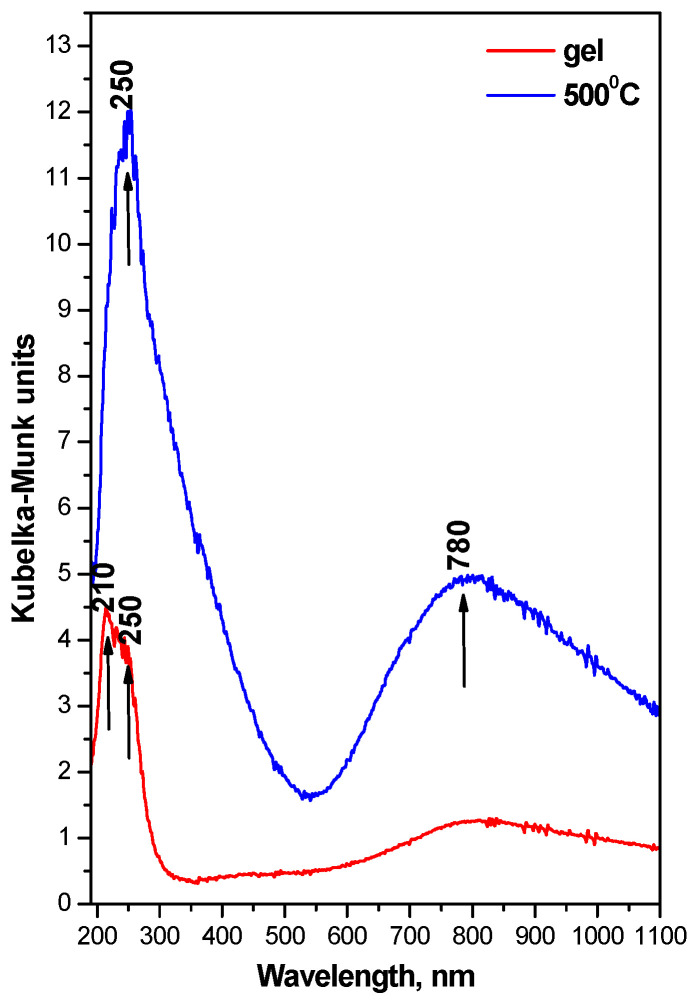
UV-Vis spectra of the investigated samples.

The UV-Vis spectra of the investigated samples are presented in Figure 3. This is one of the powerful methods to determine the chemical state and content of metal species. As is seen from the figure, both samples exhibited absorption, but the heat-treated one showed stronger absorption in the UV region. Several absorption bands centered at 210, 250, and 780 nm were observed. It is well known that the absorption peak at about 210 and 280 nm in the UV-Vis spectra of Cu-based composites can be attributed to charge transfer from lattice O^2−^ to Cu^2+^; the UV band at ~750 nm is related to the d–d transitions of Cu^2+^ species with distorted octahedral coordination. All of those bands are characteristic bands of isolated Cu^2+^ species [23,24,25]. Similar optical properties of Cu/SiO_2_ nanocomposites prepared by sol–gel processing [26] were reported, and their optical properties have been studied.

### 2.3. Antimicrobial Activity

In this study, we evaluated the antimicrobial efficacy of Si/Cu (gel) and Si/Cu/500 and nanocomposites against a range of microorganisms, including Gram-positive bacteria *Staphylococcus aureus* ATCC 25923 and *Bacillus cereus* ATCC 11778, Gram-negative bacteria *Escherichia coli* ATCC 25922, *Salmonella typhimurium* ATCC 14028, and *Pseudomonas aeruginosa* ATCC 27853, as well as the yeast species *Candida albicans ATCC 18804* and *Saccharomyces cerevisiae* CCY 21-6-3. To comprehensively assess the antimicrobial activity of the synthesized materials, we conducted both quantitative (microdilution assay in 96-well plates) and qualitative (spot test) evaluations. The spot test served as a preliminary screening method to predict the antibacterial potential of the samples. However, for this report, we present only the results derived from the quantitative analysis.

**Figure 4:**
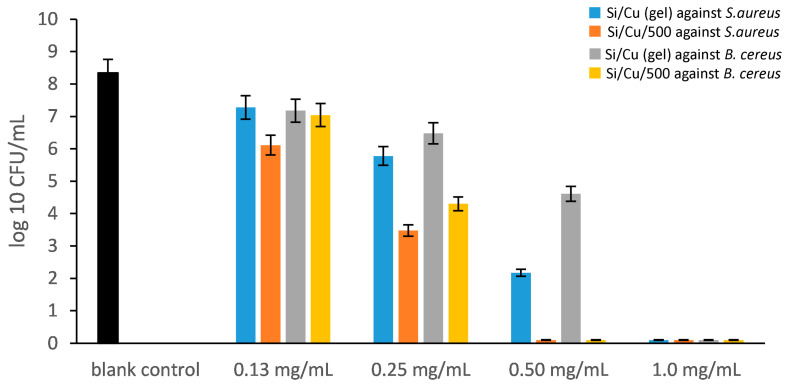
The antimicrobial activity of Si/Cu composites against *Staphylococcus aureus* ATCC 25923 and *Bacillus cereus* ATCC 11778 at the 24th hour.

As illustrated in Figure 4, both Si/Cu nanocomposites exhibited significant antibacterial activity, with their minimum inhibitory concentration (MIC) and minimum bactericidal concentration (MBC) values determined against the two Gram-positive bacterial strains. The MIC of Si/Cu (gel) against *Bacillus cereus* was found to be 0.5 mg/mL, with an MBC of 1.00 mg/mL. In contrast, the MIC of Si/Cu/500 against *B. cereus* was 0.25 mg/mL, while the MBC was 0.5 mg/mL. These findings indicate that *B. cereus* demonstrated higher sensitivity to the heat-treated Si/Cu/500 nanomaterial. A similar pattern of antibacterial inhibition was observed against the other Gram-positive bacteria, *Staphylococcus aureus* with MIC and MBC values for both nanocomposites remaining consistent (Figure 4, Figures S1 and S2). Consequently, *S. aureus* also exhibits increased susceptibility to the heat-treated nanomaterials.

**Figure 5:**
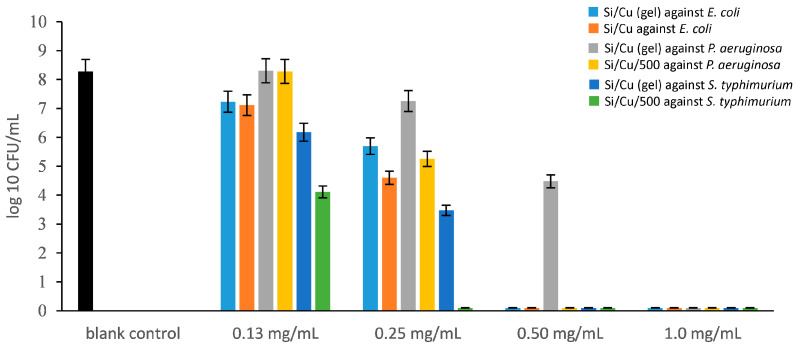
The antimicrobial activity of Si/Cu composites against *Escherichia coli* ATCC 25922, *Pseudomonas aeruginosa* ATCC 27853 and *Salmonella typhimurium* ATCC 14028 at the 24th hour.

Figure 5 (including Figures S3 and S4) presents the antibacterial activity of the two Si/Cu nanomaterials against three Gram-negative bacterial strains, as assessed by the broth microdilution method to determine the minimum inhibitory concentration (MIC) and minimum bactericidal concentration (MBC). Both nanocomposites exhibited notable inhibitory effects across the tested concentration range (from 1.00 mg/mL to 0.13 mg/mL). Among the Gram-negative strains, *Pseudomonas aeruginosa* demonstrated the highest resistance, with MIC and MBC values for the Si/Cu (gel) recorded at 0.5 mg/mL and 1.0 mg/mL, respectively. In comparison, the heat-treated material Si/Cu/500 showed enhanced efficacy, with the MBC reduced by half, indicating greater antibacterial potency against *P. aeruginosa*. *Salmonella typhimurium* was identified as the most susceptible strain, with MIC and MBC values of 0.25 mg/mL and 0.50 mg/mL, respectively, for the gel form, and 0.13 mg/mL for both MIC and MBC when treated with Si/Cu/500.

**Figure 6:**
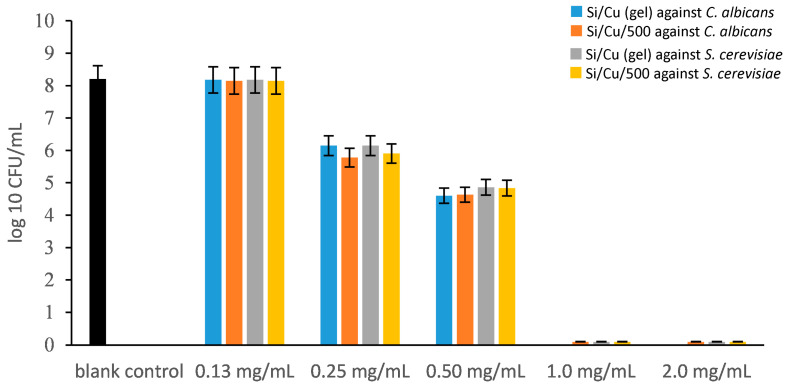
The antimicrobial activity of Si/Cu composites against *Candida albicans* ATCC 18804 and *Saccharomyces cerevisiae* CCY 21-6-3 at the 24th hour.

The final two microorganisms tested for susceptibility to the synthesized nanomaterials were the yeast species *Candida albicans* and *Saccharomyces cerevisiae*. Although yeasts are generally considered more resistant to antimicrobial agents compared to bacteria, the results obtained in this study (Figure 6) indicate comparable sensitivity to the Si/Cu nanocomposites. Both *C. albicans* and *S. cerevisiae* exhibited minimum inhibitory concentrations (MICs) of 0.5 mg/mL for both nanomaterial types, while complete fungicidal activity (MBC) was observed at a concentration of 1.0 mg/mL.

**Figure 7:**
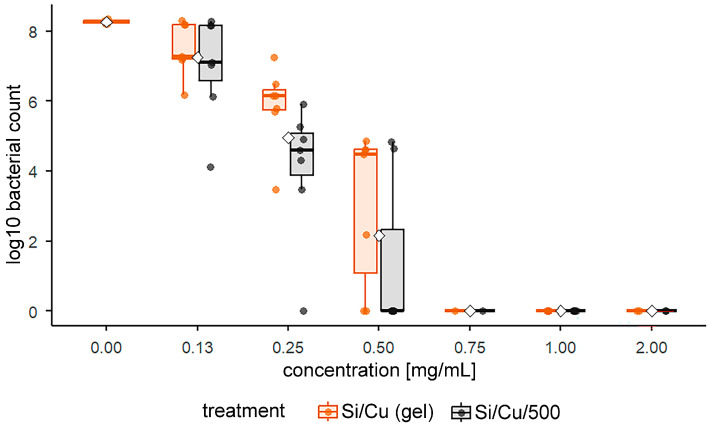
Log_10_ bacterial counts for Si/Cu (gel) and Si/Cu 500 across concentrations: the boxplots show the median and interquartile range, while the individual dots represent all measured replicates, illustrating the full data distribution; the white diamonds indicate the mean.

When comparing treatments at intermediate concentrations (Figure 7), Si/Cu/500 consistently demonstrated a stronger antimicrobial effect than the gel. At 0.25 mg/mL, cultures treated with the gel contained an average of 0.99 log_10_ higher number of bacteria (≈9.7 times more survivors) compared to Si/Cu/500 (paired *t*-test, *p* = 0.0047, n = 7), indicating a significantly weaker effect. At 0.50 mg/mL, the difference remained significant (0.92 log_10_, ≈8.4 times) but did not reach conventional statistical significance (*p* ≈ 0.096). At 0.13 mg/mL, the difference was smaller (0.27 log_10_, ≈1.9 times) and non-significant (*p* ≈ 0.145). Both materials showed a concentration-dependent decline in bacterial counts, with near-complete inhibition at ≥0.75 mg/mL.

### 2.4. Chemiluminescent Oxidation Tests

The newly synthesized nanomaterials demonstrated inhibition of the free radical and ROS oxidation in all model chemical systems. This effect can be explained by the following factors:

- The comparatively big size of the nanoparticles may favor aggregation;
- Their substantial mass, which reduces their efficient interaction with the reactive agents, despite the extensive stirring and homogenization;
- The strong stabilization of the copper ions (Cu^2+^) within the silica matrix; this could restrict their active involvement in the redox reactions, mostly affecting the Fenton’s reaction.

Although copper is naturally highly reactive and the release of Cu^2+^ ions would typically enhance oxidative reactions, the registered chemiluminescence remained consistently lower than that of the control reactions, lacking nanomaterials. Thus, the effect of these hybrids is more accurately characterized as inhibitory towards the free radical-driven oxidation, rather than traditionally antioxidant, with respect to the electronic interactions of silicon and copper within the composite matrix.

The confirmed safety and biocompatibility of the materials support their potential use in diverse applications, including:

- Drug delivery platforms designed to achieve controlled release of therapeutics within the biological system;
- Biomaterials designed for implantable devices or tissue-engineered constructs, wherein the mitigation of oxidative stress and prevention of cellular damage are very important;
- Surface-modifying agents at low concentrations, offering antibacterial functionality without cytotoxic effects.

**Figure 8:**
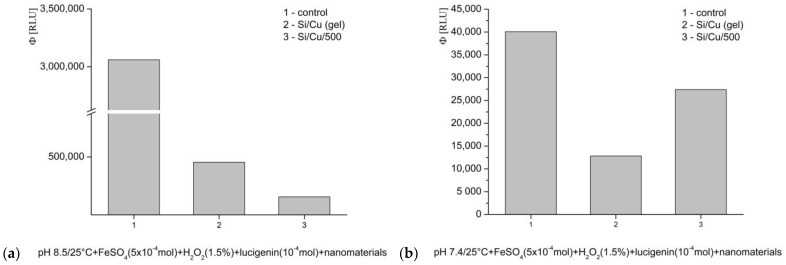
The effect of Si/Cu (gel) and Si/Cu/500 nanocomposites on luminescence, presented as quantum yields, in a system generating ·OH and ·OOH radicals, at pH 8.5 (**a**) and pH 7.4 (**b**).

The tested samples showed the following results about the tested modeled systems:

Fenton’s system, generating ·OH and ·OOH radicals: at pH 8.5 (optimal), the tested nanocomposites demonstrated an inhibitory effect. Si/Cu (gel) decreased luminescence by 85%, Si/Cu/500—95% (Figure 8a). The same interaction was measured at pH 7.4—physiological conditions for bacteria and the internal fluid environment of the human organism. Inhibition of the signal was also observed. Si/Cu (gel) lowered the emission almost 70% and Si/Cu/500 more than 30% (Figure 8b). Si/Cu/500 presented a strong prooxidant effect in comparison to Si/Cu (gel).

This may be due to structural transformations as a result of the thermal treatment, the formation of crystalline and catalytically active copper oxide phases that could intensify the generation of ROS in a Fenton-like reaction, and the subsequently enhanced redox potential.

**Figure 9:**
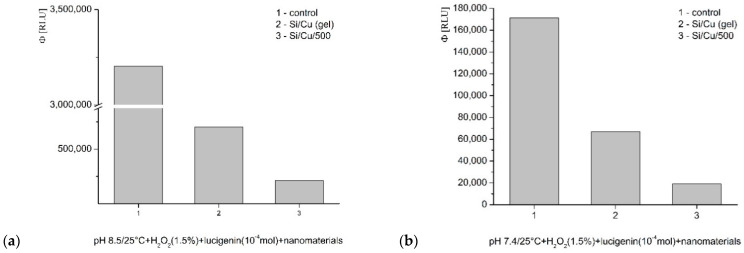
The effect of Si/Cu (gel) and Si/Cu/500 nanocomposites on luminescence, presented as quantum yields, in a system with H_2_O_2_, at pH 8.5 (**a**) and pH 7.4 (**b**).

In the second model system, H_2_O_2_ appears as a strong oxidant and an ROS. The registered inhibition at pH 8.5 was almost 80% about Si/Cu (gel) and more than 90% about Si/Cu/500 (Figure 9a). At pH 7.4, the presented inhibition by Si/Cu (gel) was not that strong—more than 60%, Si/Cu/500 confirmed almost 90% suppression of the signal (Figure 9b).

The first two oxidation model systems are indicative of the process of Cu^2+^ release that could increase the oxidation and the chemiluminescent signal above the blank control level. In both testing model chemical systems, Cu^2+^ could interact with H_2_O_2_. But they do not appear to be strong chemical interaction competitors to Fe^2+^ in the Fenton’s system; they are also not spatially or thermodynamically competitive, despite the active high applied concentrations of the tested nanohybrid materials (1 mg/mL).

**Figure 10:**
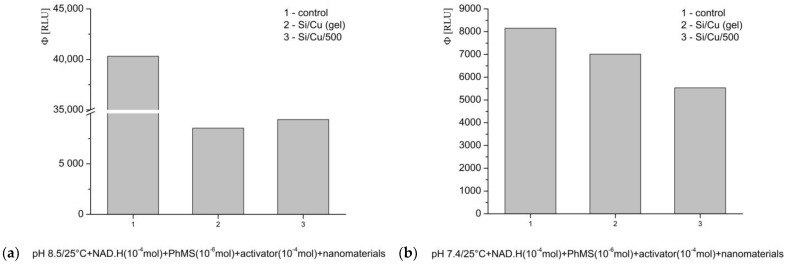
The effect of Si/Cu (gel) and Si/Cu/500 nanocomposites on luminescence, presented as quantum yields, in a system generating O_2_^·−^ radicals at pH 8.5 (**a**) and pH 7.4 (**b**).

The third tested model system is specialized for the generation of superoxide (O_2_^·−^) radicals. pH 8.5 favors strong oxidation. In these conditions both of the tested newly synthesized nanocomposites strongly decreased the registered light emission, Si/Cu (gel) —almost 80%; Si/Cu/500—more than 76% (Figure 10a). At pH 7.4, (physiological), the registered effect was lighter, with yields close to the control level (Figure 10b); Si/Cu (gel) —up to 15%, whereas Si/Cu/500—more than 30%.

In summary, the chemiluminescent assay results demonstrate that the tested newly synthesized nanocomposites exert a pronounced general inhibitory effect on the ROS generation and oxidation across the tested model systems. These findings emphasize the necessity of carefully tailoring the nanostructure with respect to the medium and intended application. The synthesized nanohybrids can be potentially applied as modulators or inhibitors of free radical processes and generation within biological systems.

### 2.5. Cytotoxicity Evaluations

**Figure 11:**
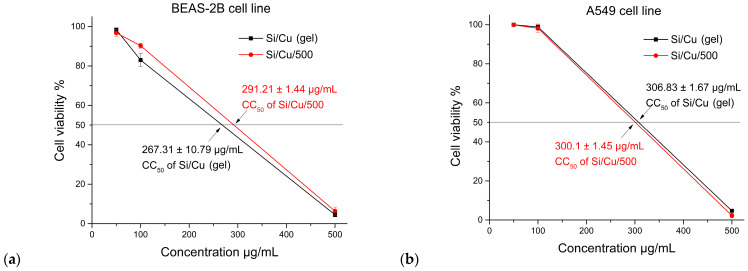
Evaluation of cytotoxicity of Si/Cu (gel) and Si/Cu/500 nanoparticles in BEAS-2B (**a**) and A549 (**b**) cells. Means and standard deviations SD of two independent experiments.

**Figure 12:**
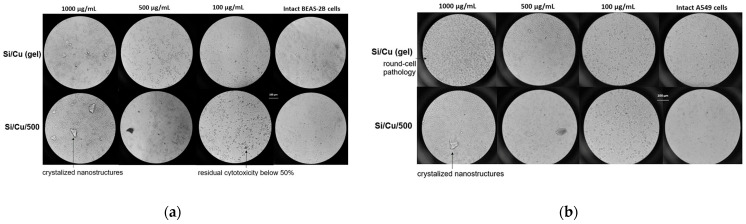
BEAS-2B (**a**) and A549 (**b**) morphology of cells treated with Si/Cu (gel) and Si/Cu/500 nanoparticles under the inverted light microscope (Olympus CK40, magnification 20×).

CuSO_4_ is used to control diseases and algae, for instance, in the aquaculture industry [27]. The toxicity of CuSO_4_ to fish and other animals, including humans, is relatively well known, including tissue damage and oxidative stress [28]. Copper is an essential trace element, but it also exerts cytotoxic effects through the induction of ROS release. Copper sulfate was found to exert cytotoxic effects in human HeLa, endometrial (HEC-1-A) and lung (A549) adenocarcinoma cells, but not in normal human kidney (HEK293) or bronchial (Beas-2B) epithelial cells [29]. Here, we have evaluated the cytotoxic effects of Si/Cu (gel) and Si/Cu/500 nanocomposites on the cell viability of human normal (BEAS-2B) and tumor epithelial (A549) cells, with microscopic recordings of the morphological alternations induced at various concentrations (Figure 11). No significant differences were found between the two nanomaterials; the calculated CC_50_ values were similar for the tumor and non-tumor cells, being in the range 267.31–306.83 µg/mL (Figure 11 and Figure 12). According to the literature data, regarding the tumor type of cells, CuSO_4_ has CC_50_ 255–300 µM (39.9–47.9 µg/mL), which reveals the nanomaterials studied as much less toxic. Our results differ from other studies’ data, showing higher susceptibility of cancerous cells to Cu-based materials [30,31]. A possible explanation could be the immortalization of normal bronchial BEAS-2B line and presence of FBS in the medium, which promotes the cells to high rates of proliferation and metabolic activity. Another point to consider is the poor solubility of materials tested and the potential accidental occurrence of non-dissolved crystals, releasing Cu ions in the culture medium and raising their concentrations during the 48 h of incubation.

### 2.6. Daphnia magna Tests

**Figure 13:**
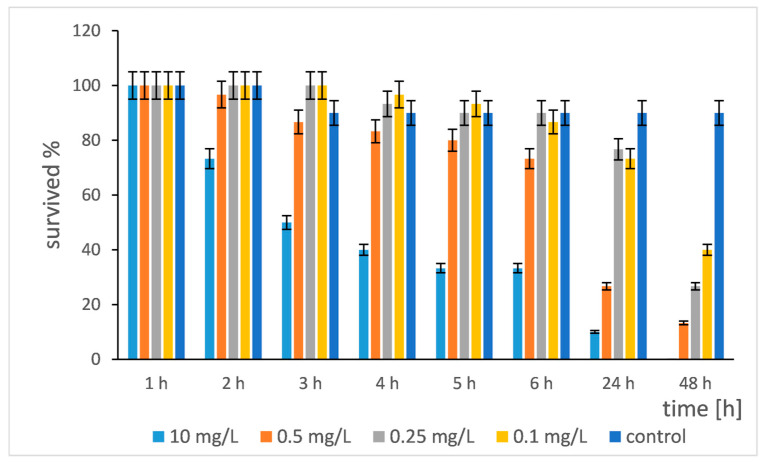
*Daphnia magna* survival rate after treatment with Si/Cu (gel) nanocomposites.

The first toxicological test was conducted with daphnia (*Daphnia magna*) exposed to different concentrations of Si/Cu (gel) for a period of 48 h. The test tracked the survival rate of daphnia over time (1, 2, 3, 4, 5, 6, 24 and 48 h) at four different concentrations (10 mg/L, 0.5 mg/L, 0.25 mg/L, 0.1 mg/L) in three replicates and two controls (untreated daphnia) (Figure 13).

The figure presents the survival rate of *Daphnia magna* for 48 h upon exposure to different concentrations of Si/Cu (gel) nanocomposites. The results showed a clearly expressed “concentration–effect” relationship: the higher the concentration, the faster and more serious the survival rate decreased. At the highest concentration (10 mg/L) the survival rate dropped dramatically to 73.3% after only 1 h and reached 0% after 48 h. This indicated very high toxicity at this concentration. At decreasing concentrations of 0.5 mg/L, 0.25 mg/L and 0.1 mg/L, it was seen that when the concentration decreased, the effect slowed down and declined. At 0.5 mg/L, there was still significant mortality (13.3% survived after 48 h). At 0.25 mg/L, the reduction was slower, but after 48 h, the survival rate was still significantly reduced (26.7%). The lowest concentration was 0.1 mg/L. This was the least affected experimental group. After 48 h, survival was 40%, which indicated that even low concentrations had acute toxic effect. The time dynamics showed that the most drastic changes occurred during the first 6 h, especially at higher concentrations. That indicated an acute toxic effect. During the period from 6 to 48 h, the rate of survival decrease slowed down, with a significant part of the mortality occurring. That also indicated a prolonged effect. The control group had a stable survival of 90% throughout the experiment. The slight decrease from 100% to 90% in the first hours may be due to normal stress from the manipulation or natural mortality in the population. The stability of the control proved that the decrease in survival in the experimental groups was a result of the effect of Si/Cu (gel) and was not due to other factors.

The data allowed an approximate estimate of the lethal concentration (LC_50_). After 24 h, LC_50_ was between concentrations of 0.5 mg/L (73.3% survival and 26.7% mortality) and 0.25 mg/L (90% survival and 10% mortality). Therefore, the LC_50_ (24 h) was probably close to 0.31 mg/L. After 48 h, the LC_50_ was between concentrations of 0.25 mg/L (26.7% survival) and 0.1 mg/L (40% survival). Therefore, the LC_50_ (48 h) was lower than 0.25 mg/L, probably around 0.15–0.2 mg/L. That showed that the increasing exposure time increased toxicity.

The results obtained for the toxicity of Si/Cu (gel) to *Daphnia magna* show a clear acute toxicity starting in the first hours and lower LC_50_ values later (24–48 h), which is consistent with data from other studies on copper nanomaterials. For example, Thit et al. (2017) reported that CuO nanoparticles can cause significant mortality at concentrations below 1 mg/L, with LC_50_ (48 h) decreasing in the presence of better dispersion or smaller particles [32]. Similar results were reported by Santos-Rasera et al. (2019), who found extremely high toxicity of small CuO nanoparticles (25 nm), with LC_50_ (48 h) around 0.05 mg/L [19].

The comparison shows that the LC_50_ values reported in the present study (24 h ≈ 0.31 mg/L; 48 h ≈ 0.15–0.20 mg/L) are in the range of the more toxic Cu-containing nanomaterials published in the literature. This suggests that copper incorporated into the Si/Cu (gel) is likely bioavailable and exhibits an acute toxic effect similar to that described by Cojocaru et al. (2025), according to which copper ions and nanoforms at low concentrations can cause rapid mortality in *Daphnia magna* [33]. The observed rapid decline in survival during the first 1–6 h is also consistent with the characteristic dynamics described in the study by Thit et al. (2017) [32], in which highly dispersed Cu nanoparticles lead to an early phase of acute toxicity, followed by a slower chronic phase. In this context, the data obtained from Figure 13 confirm that the Si/Cu (gel) nanocomposite has an acute, concentration-dependent toxic effect, comparable to that of free Cu or CuO nanoparticles [32].

**Figure 14:**
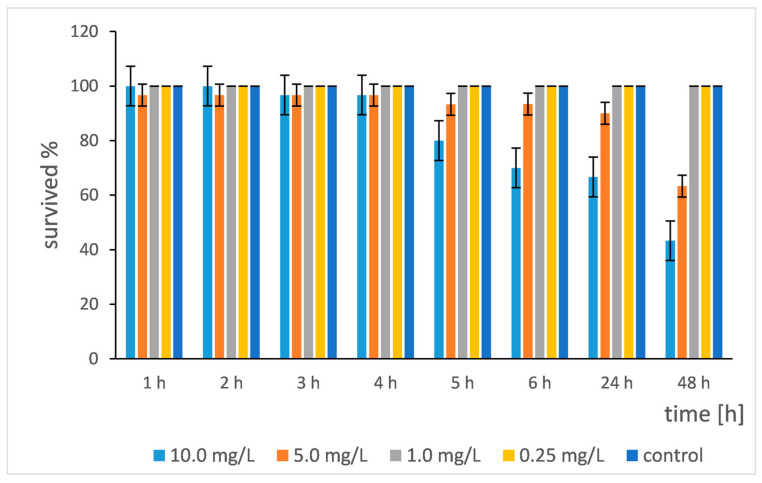
*Daphnia magna* survival rate after treatment with Si/Cu/500 nanocomposites.

Figure 14 shows a clear “dose–response relationship”: the higher the concentration, the stronger the lethal effect and the faster it occurred. At the highest concentration (10.0 mg/L), the effect was very fast and strong. Mortality was observed after 3 h (96.7% survival, i.e., 3.3% mortality). After 48 h, survival dropped to 43.3%, which meant that more than half of all daphnia (56.7%) died. At a concentration of 5.0 mg/L, the effect was slower. Noticeable mortality occurred only after 24 h (90% survival). After 48 h, mortality was 36.7%. At low concentrations (1.0 mg/L and 0.25 mg/L), no mortality was observed during the entire 48 h period. Survival remained 100%, the same as in the control group.

Regarding the time–response relationship, the results showed that for higher concentrations, the effect of nanoparticles increased with time. At 10.0 mg/L, there was a constant trend of decreasing survival. At 5.0 mg/L, a significant decrease was seen between the 24th and 48th hours.

Important toxicological indicators have been derived from the data. It was found that the LC_50_ for 48 h was between 5.0 mg/L and 10.0 mg/L. At 5.0 mg/L, the mortality was 36.7%, and at 10.0 mg/L, it was 56.7%. Therefore, the LC_50_ was approximately 8.84 mg/L. The NOEC (No Observed Effect Concentration) was 1.0 mg/L (since there was no effect at 1.0 mg/L and 0.25 mg/L). The LOEC (Lowest Observed Effect Concentration) was 5.0 mg/L (since mortality was observed at that concentration). The control group had 100% survival throughout the experiment. This was critically important because it confirmed that the mortality in the other groups was indeed due to the action of the toxicant, and not to the stress from the experimental conditions (e.g., starvation, poor water quality).

Si/Cu/500 was toxic to daphnia at concentrations of 5.0 mg/L and higher. The toxicity was time- and concentration-dependent. The effect was faster and stronger with the increase in the dose. The threshold of observable toxicity for this 48 h test was between 1.0 mg/L and 5.0 mg/L. Concentrations of 1.0 mg/L and lower did not show a lethal effect within 48 h under the chosen conditions.

The results obtained for Si/Cu/500 show a significantly lower acute toxicity to *Daphnia magna* compared to classical copper nanoparticles, which is consistent with the data in the literature. While in the present study, the LC_50_ (48 h) is approximately 8.84 mg/L, most studies on CuO or Cu nanoparticles report much higher acute toxicity. For example, Santos-Rasera et al. (2019) found an LC_50_ (48 h) of around 0.1 mg/L for CuO nanoparticles, which is over 80 times lower than that observed for Si/Cu/500 [19]. Similar values were also reported by Kien et al. (2017), who measured LC_50_ in the range of 0.05–0.2 mg/L, indicating an extremely high bioavailability of free copper nanoparticles [34].

Additionally, Arratia et al. (2019) compared Cu nanoparticles with their microparticles and showed that the nanoforms resulted in early and severe mortality at concentrations below 1 mg/L—a much stronger effect than the one of Si/Cu/500 [35]. The study by Saif et al. (2016) on naturally synthesized and engineered CuO particles also showed that both types of materials caused acute toxicity at concentrations below 0.5 mg/L [36].

In this context, the higher LC_50_ values and the lack of effect at low concentrations (1.0 and 0.25 mg/L) presented in Figure 14 suggest a significantly reduced bioavailability of copper in Si/Cu/500 likely due to stabilization in the Si matrix, reduced dissolution, or limited migration of Cu^2+^ ions. This is consistent with the trend that composite or matrix-stabilized metal nanomaterials exhibit lower acute toxicity compared to “naked” nanoparticles, as also described by Santos-Rasera et al. (2019) and Arratia et al. (2019) [19,35].

### 2.7. Hypotheses and Conclusions About the Observed Different Effects Between Si/Cu (Gel) and Si/Cu/500

- Antimicrobial effects—the toxicity of the newly synthesized nanocomposites is reduced; Si/Cu/500 appeared more toxic to all tested microorganisms, probably due to its higher reactive surface.
- The thermal treatment of Cu/Si/500 nanocomposite decreased the availability of free copper ions, stabilizing them in oxide or silicate lattices, and thus reducing their acute toxicity to daphnia, usually very sensitive to dissolved metal ions.
- According to the ROS activity chemiluminescent tests, both tested nanocomposites inhibited the ROS generation. Si/Cu/500 inhibited the generation of ROS strongly and presented weaker oxidation activity in comparison to Si/Cu (gel). Only at pH 7.4, in the Fenton’s system, Si/Cu/500 presented higher prooxidant activity in comparison to Si/Cu (gel). That may probably be due to Cu^2+^ release from the thermally crystalline-shaped structure and induced higher redox potential in a Fenton-like reaction.
- Both Si/Cu (gel) and Si/Cu/500 nanocomposites exhibited comparable cytotoxicity, inducing similar morphological alterations and CC_50_ values in human normal and tumor cells. Their effects suggest moderate cytotoxicity.

## 3. Materials and Methods

### 3.1. Materials and Preparation of the Gels

The silica–copper gel was synthesized using the sol–gel method with a nominal composition of 95SiO_2_/5CuO (mol%). The reagents employed were tetraethyl orthosilicate (TEOS) and C_2_H_5_OH by Sigma-Aldrich, Burlington, MA, USA, and CuSO_4_·5H_2_O by Fluka Chemie AG, Buchs, Switzerland. For solution preparations, silicon alkoxide was added under continuous stirring to 100 mL of absolute ethanol containing a pre-dissolved amount of CuSO_4_·5H_2_O. Subsequently, water was introduced at a molar ratio of H_2_O/TEOS = 1:4 to initiate the hydrolysis and polycondensation of the Si–(OEt) groups. The pH was adjusted to approximately 3 using a few drops of HCl. Gelation occurred at room temperature within about 10 h. The obtained gel was then heat-treated at 500 °C for 3 h, resulting in dark-colored materials. The prepared samples were designated as Si/Cu (gel) and Si/Cu/500 (heat-treated at 500 °C).

### 3.2. Sample Characterization

Powder X-ray diffraction (XRD) measurements were carried out at room temperature using a Bruker D8 Advance diffractometer (Berlin, Germany) equipped with Cu Kα radiation (λ = 1.54056 Å) and a LynxEye position-sensitive detector. The X-ray source was operated at 40 kV and 40 mA. Diffraction data were collected over a 2θ range of 5.3–80° 2 h with a step size of 0.02° 2 h. Surface morphology was examined by scanning electron microscopy (SEM) using a HIROX SH-5500 instrument (Hirox Japan Co., Ltd., Tokyo, Japan), coupled with a QUANTAX 100 Advanced energy-dispersive X-ray spectroscopy (EDS) system (Bruker Co., Frankfurt, Germany). The samples were studied as powders. Before the analysis, the surface of the samples was coated with a thin layer of gold. The optical absorption spectra of the powdered samples were recorded using a UV–Vis diffuse reflectance spectrophotometer (Evolution 300, Thermo Electron Corporation, Madison, WI, USA). Magnesium oxide was employed as a reflectance standard for baseline correction, and the measurements were performed over the wavelength range of 200–1100 nm.

### 3.3. Materials Used for the Antimicrobial Activity Testing

The microbial strains applied in this study were obtained from the National Bank for Industrial Microorganisms and Cell Cultures (NBIMCC, Sofia, Bulgaria). The testing panel comprised two Gram-positive bacteria—*Staphylococcus aureus* ATCC 25923 and *Bacillus cereus* ATCC 11778—and three Gram-negative bacteria—*Salmonella typhimurium* ATCC 14028, *Escherichia coli* ATCC 25922, and *Pseudomonas aeruginosa* ATCC 27853. In addition, two yeast strains, *Saccharomyces cerevisiae CCY 21-6-3* and *Candida albicans ATCC 18804*, were selected as representative eukaryotic commensals. Before use, the synthesized nanoparticles were dispersed via sonication for 1 h using a Sonoplus ultrasonic processor (Berlin, Germany) to prevent agglomeration and ensure homogenous distribution. Serial dilutions of the nanomaterials were then prepared in an appropriate solvent to cover a concentration range consistent with reported antimicrobial activity. To initiate antimicrobial testing, fresh bacterial cultures were prepared by subculturing overnight-grown colonies on agar plates into appropriate liquid media to achieve mid-logarithmic phase growth. The resulting bacterial suspensions were standardized to a turbidity equivalent to 0.5 McFarland standard (approximately 1–2 × 10^8^ CFU/mL). For the microdilution assay, 100 µL of the proper concentration of the nanoparticles in Mueller–Hinton broth was dispensed into each well of a sterile 96-well microtiter plate. Serial dilutions of the test compounds were added to the wells, followed by the addition of 100 µL of the standardized microbial suspension to achieve a final inoculum concentration of approximately 5 × 10^7^ CFU/mL per well. Plates were incubated at 36 ± 1 °C for 20–24 h.

Following incubation, wells were visually inspected for microbial growth for minimum inhibitory concentration (MIC), and from the corresponding concentrations that showed an inhibitory effect, serial descending dilutions were made to determine the minimum bactericidal concentration (MBC). The MIC is defined as the lowest concentration of an antimicrobial agent that prevents visible microbial growth, whereas the MBC represents the lowest concentration required to achieve complete bacterial killing. These parameters provide insights into whether the tested agents exert bacteriostatic or bactericidal effects, thereby informing their mode of action.

After incubation, bacterial growth was assessed, and colony-forming units (CFUs) were enumerated to determine MBC values. Negative controls, consisting of bacterial suspensions without exposure to antimicrobial agents, were included in all assays to validate the experimental conditions. All experiments were conducted in triplicate, and the data are presented as mean values ± standard deviation (SD) derived from three independent biological replicates (n = 3).

### 3.4. Chemiluminescent Assay

The activated chemiluminescence assay can measure the concentration of the products (free radicals and ROS) in reference luminescent units (RLU) as well as the kinetics of the reaction using minimum volumes. It provides dynamic insight into the radical-driven reactions; it allows evaluation of the prooxidant and antioxidant/inhibitory potential, thereby clarifying whether the tested sample exerts harmful or protective effects. The naturally weak emission produced in such metabolic reactions can be strongly enhanced by the application of physical or chemical activators (probes). The automated recording of the measured data ensures real-time registration of the reactions’ kinetics, followed by calculations of the quantum yields, the rate activation/inhibition constants, and some other parameters in the interactions with the examined compounds.

We applied a final active concentration of the nanocomposites of 1 mg/mL; it is considered relatively high. The luminescent response was compared with that of a control reaction lacking nanomaterials (blank). The influence of the newly synthesized hybrids on the kinetics and generated products of the free radical oxidation was tested ex vivo at physiological pH 7.4 and pH 8.5, which favors radical generation, at 25 °C, applying the lucigenin-activated chemiluminescence by the following model systems:

(1) Fenton’s system (H_2_O_2_–FeSO_4_) for the generation of hydroxyl (·OH) and hydroperoxyl (·OOH) radicals;

(2) System with hydrogen peroxide (H_2_O_2_);

(3) (NAD.H–phenazine methosulfate) system, for the generation of superoxide radicals (O_2_^·−^).

All measured data were statistically processed. The significant effects were presented as quantum yields, which are calculated integral values, representing the registered effects.

Fenton’s system: The reaction mixture comprised of 0.2 mol sodium hydrogen phosphate buffer adjusted to the chosen pH, Fenton’s reagent consisting of FeSO_4_ at 5 × 10^−4^ mol concentration and H_2_O_2_ (1.5%) plus the chemiluminescent probe lucigenin at 10^−4^ mol. ROS and free radicals were produced in the system along the following reaction mechanism:

Fe^2+^ + H_2_O_2_ → Fe^3+^ + ^·^OH+ ^−^OH

Fe^3+^ + H_2_O_2_ → Fe^2+^ + ^·^OOH + H^+^

System with hydrogen peroxide (H_2_O_2_): The preparation included a 0.2 mol sodium hydrogen phosphate buffer set to the chosen pH value, along with H_2_O_2_ (1.5%) plus the chemiluminescent probe lucigenin at 10^−4^ mol. Here, hydrogen peroxide functions simultaneously as an oxidizing agent and an ROS.

NAD.H–phenazine methosulfate system: The sample comprised of 0.2 mol sodium hydrogen phosphate buffer adjusted to the chosen pH, NAD.H at 10^−4^ mol concentration, phenazine methosulfate at 10^−6^ mol, plus the chemiluminescent probe lucigenin at 10^−4^ mol. The generation of superoxide radicals in this chemical system proceeds according to the following reaction scheme:

PhMS + NAD.H + H^+^ → PhMS.H_2_ + NAD^+^

PhMS.H_2_ + PhMS → 2 PhMS.H^.^

PhMS.H^.^ + O_2_ → PhMS + O_2_^.−^ + H^+^

All systems and every reaction were tested for 3 min, every 3 s, measured by LUMIstar Omega (BMG Labtech GmbH, Ortenberg, Germany, 2020). All nanomaterials were sonicated for at least 60 min before the test at 25 °C. All materials were used “fresh”—very soon after their production—in order to prevent oxidation, humidification, and agglomeration. Additional vortex and shaking procedures were applied in order to ensure reproducibility. All experiments were performed in triple reproducible measurements. The statistical analysis was performed by Origin 8.5 and Microsoft Office Excel 2010, and Student’s *t*-test, *p* ≤ 0.05.

### 3.5. Cytotoxicity Assay

- Compounds

Si/Cu (gel) and Si/Cu/500 nanoparticles were dissolved at the beginning of each experiment in Dulbecco MEM maintenance medium (DMEM), as they were suspended and ultrasonicated for 15 min before the serial dilutions within the range 1–1000 μg/mL.

- Cell lines

BEAS-2B (ATCC-CRL-3588™) normal human bronchial epithelial cells, derived from autopsies of noncancerous individuals and human lung carcinoma A549 (ATCC-CCL-185™) cells were cultured at 37 °C in a 5% CO_2_ incubator Thermo Forma 310 (Thermo Fisher Scientific, Waltham, MA, USA), as adherent cultures in the relevant plasticware (Costar^®^, Corning, NY, USA) and DMEM growth medium (Gibco, Grand Island, NY, USA) supplemented with 10% FBS (Gibco), 3.7 mg/mL sodium bicarbonate, 10 µM HEPES buffer (AppliChem GmbH, Darmstadt, Germany) and antibiotics. Cytotoxicity testing was performed on a minimum of 90% confluent cell monolayers at a density of 2.5 × 10^5^/mL, with microscopic and spectrophotometric detection 48 h after the treatment.

- Determination of cytopathic effects

The presence of cytotoxic effects was registered 48 h after the incubation with varying concentrations of the substances under a light inverted microscope (Olympus CK 40, Tokyo, Japan) and photographed at ×20 magnification. The natural red (NR) uptake assay was conducted and the cytotoxic concentration 50% (CC_50_) was calculated, as described previously [13].

- Statistical Analysis

Testing was performed in duplicates, with four wells being treated with each concentration within one trial. The results were analyzed using Biotek Organon microplate reader (West Chester, PA, USA) with Gen5^®^ 3.04 and Excel^®^ MS Office 2021 software. The graphs were prepared using Origin 8.5^®^.

### 3.6. Daphnia magna Toxicity Test

Standardized tests were used to conduct the toxicological studies (Test No. 202: *Daphnia* sp. acute immobilization test) [37].The toxicological tests were conducted with daphnia (*Daphnia magna*) exposed to different concentrations of the tested nanoparticles for a period of 48 h. The test monitored the survival rate of daphnia over time—from 1 to 48 h, at four different concentrations (10.0 mg/L, 5.0 mg/L, 1.0 mg/L and 0.25 mg/L), in three replicates and controls—untreated daphnia.

## 4. Conclusions

Si/Cu-hybrid nanomaterials were prepared by a sol–gel method. The SEM-EDS analysis proved the presence of Si, Cu and O elements.

When comparing treatments with intermediate concentrations, Si/Cu/500 (calcined at 500 °C) consistently demonstrated a stronger antimicrobial effect compared to the gel form nanomaterial (Si/Cu (gel)). The tested nanomaterials had similar antimicrobial effects on different microorganisms. The small difference was proved for low concentrations of 0.25 mg/mL, but at higher concentrations there was no significant difference between the heated and gel materials.

The luminescent study revealed that the tested newly synthesized nanocomposites strongly suppressed ROS generation and oxidation across the different chemical model systems. The effect was strongly dependent on pH. These observations highlight the importance of careful selection of the type of matrix according to the intended application. The presented nanohybrids could act as regulators and inhibitors of free radical processes in biological systems, including living eukaryotic systems. These results were also confirmed by the cytotoxicity evaluations. Both Si/Cu (gel) and Si/Cu/500 nanocomposites exhibited comparable cytotoxicity, inducing similar morphological alterations and CC_50_ values in human normal and tumor cells. Their effects suggest moderate cytotoxicity.

Si/Cu (gel) was toxic to daphnia even at relatively low concentrations. Toxicity was time and concentration-dependent: higher concentrations resulted in faster mortality. *Daphnia* are a suitable bioindicator for assessing the toxicity of these new nanoproducts in the aquatic environment. The obtained information is critically important for assessing the ecological risk in water systems and for determining safe accumulation limits.

## Data Availability

The original contributions presented in this study are included in the article and Supplementary Material. Further inquiries can be directed to the corresponding author.

## Supplementary Materials

The following supporting information can be downloaded at: https://www.mdpi.com/article/10.3390/ph19010035/s1, Figure S1: *Staphylococcus aureus* influenced by Si/Cu (gel) in a concentration of 0.13 mg/mL; Figure S2: *Staphylococcus aureus* influenced by Si/Cu (gel) in a concentration of 0.13 mg/mL in the 4th consecutive decimal dilution; Figure S3: *Escherichia coli* after inhibition by Si/Cu (gel) in a concentration of 0.25 mg/mL; Figure S4: *Escherichia coli* after inhibition by Si/Cu (gel) in a concentration of 0.25 mg/mL in the 3rd consecutive dilution.
