# Supplementary material for: Antibacterial, Redox, Cytotoxic, and Ecotoxic Properties of New Sol–Gel Silica-Copper-Based Materials
Source: Pharmaceuticals (Basel). 2025 Dec 23;19(1):35. doi: 10.3390/ph19010035 (PMC12844963; doi:10.3390/ph19010035)
Supplement: Supplementary file 1 [file pharmaceuticals-19-00035-s001.zip › pharmaceuticals-4025470-supplementary.pdf]

SUPPLIMENTARY MATERIAL

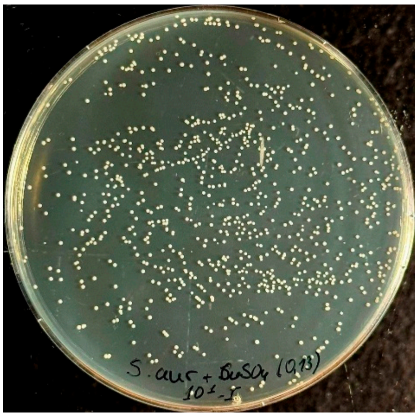

Figure S1. *Staphylococcus aureus* influenced by Si/Cu (gel) in a concentration of 0.13 mg/ml

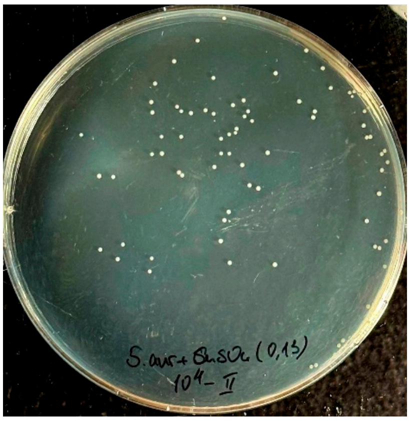

Figure S2. *Staphylococcus aureus* influenced by Si/Cu (gel) in a concentration of 0.13 mg/ml in the 4th consecutive decimal dilution

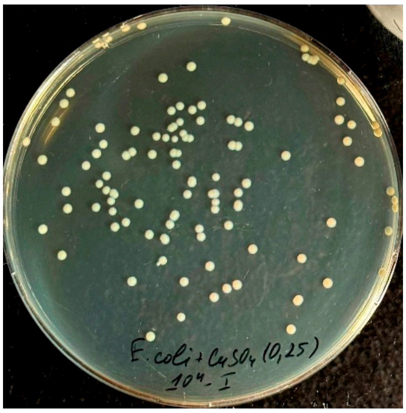

Figure S3. *Escherichia coli* after inhibition by Si/Cu (gel) in a concentration of 0.25 mg/ml

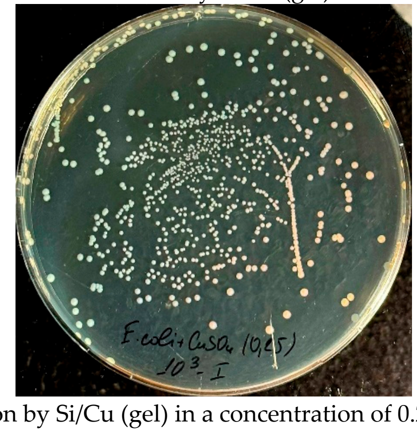

Figure S4. *Escherichia coli* after inhibition by Si/Cu (gel) in a concentration of 0.25 mg/ml in the 3rd consecutive dilution
